# Supplementary material for: Pain in Multiple Sites and Clusters of Cause-Specific Work Disability Development among Midlife Municipal Employees
Source: Int J Environ Res Public Health. 2021 Mar 24;18(7):3375. doi: 10.3390/ijerph18073375 (PMC8037270; doi:10.3390/ijerph18073375)
Supplement: Supplementary file 1 [file ijerph-18-03375-s001.pdf]

Supplementary materials

Supplementary table 1: Predictors of the work disability clusters. Odds ratios (using none work disability cluster as reference group) and their 95 % confidence intervals [95% CI]. N=2,878. Missing values imputed.

	Work disability clusters (ref. the none work disability cluster 1)			
	**2. Minor various**	**3. Mental**	**4. Musculoskeletal**	**5. Other type**
	OR [95% CI]	OR [95% CI]	OR [95% CI]	OR [95% CI]
**Pain (ref. no pain)**				
Single location pain	1.44**	1.01	2.74***	2.09**
	[1.15 – 1.80]	[0.50 – 2.05]	[1.80 – 4.17]	[1.20 – 3.66]
2 locations	1.65***	0.67	4.24***	3.08***
	[1.26 – 2.16]	[0.25 – 1.77]	[2.75 – 6.54]	[1.72 – 5.50]
3–7 locations	1.83***	3.34***	4.87***	3.73***
	[1.36 – 2.47]	[1.81 – 6.13]	[3.12 – 7.60]	[2.08 – 6.67]
**Common mental disorders (Ref. no)**				
Yes	1.10	2.85***	1.49*	1.85**
	[0.90 – 1.35]	[1.71 – 4.77]	[1.06 – 2.10]	[1.18 – 2.89]
**Gender (ref. men)**				
Women	1.20	1.30	1.70*	0.98
	[0.96 – 1.51]	[0.64 – 2.67]	[1.01 – 2.86]	[0.56 – 1.71]
**Age in 2007 (ref. 45-59)**				
50–44	1.19^{+}	2.23*	1.36	1.78*
	[0.98 – 1.45]	[1.20 – 4.15]	[0.93 – 1.98]	[1.06 – 3.00]
55–57	1.14	2.08*	1.62*	1.82*
	[0.93 – 1.40]	[1.08 – 3.99]	[1.12 – 2.37]	[1.07 – 3.10]
**Occupational class (ref. managers or professional)**				
Semi-professionals	1.29*	2.63**	1.61^{+}	1.94*
	[1.04 – 1.61]	[1.38 – 4.99]	[0.95 – 2.74]	[1.03 – 3.64]
Routine non-manual workers	1.69***	2.08*	4.00***	2.50**
	[1.35 – 2.12]	[1.03 – 4.17]	[2.49 – 6.44]	[1.34 – 4.66]
Manual workers	2.07***	2.87*	4.26***	4.76***
	[1.48 – 2.89]	[1.12 – 7.38]	[2.34 – 7.75]	[2.29 – 9.90]
**Working conditions**				
Physically strenuous work (ref. no)	1.01	0.75	1.95***	1.34
	[0.82 – 1.25]	[0.41 – 1.37]	[1.39 – 2.74]	[0.83 – 2.15]

Mentally strenuous work (ref. no)	1.18	2.34**	1.08	1.63+
	[0.91 – 1.53]	[1.29 – 4.21]	[0.68 – 1.72]	[0.93 – 2.84]
**Body weight (ref. BMI<25)**				
Overweight	1.22*	1.71+	1.55*	1.97**
	[1.01 – 1.46]	[0.98 – 2.99]	[1.11 – 2.17]	[1.24 – 3.11]
Obesity	1.40**	2.81**	1.84**	2.00*
	[1.09 – 1.79]	[1.51 – 5.20]	[1.21 – 2.79]	[1.13 – 3.55]
**Smoking (ref. never)**				
Past smoking	1.13	0.93	1.32	1.30
	[0.93 – 1.39]	[0.50 – 1.73]	[0.90 – 1.93]	[0.76 – 2.22]
Smoking	1.48**	1.72+	2.12***	2.84***
	[1.17 – 1.88]	[0.93 – 3.20]	[1.44 – 3.11]	[1.72 – 4.68]
**Binge drinking (once a month or more, ref. no)**	1.26*	0.82	1.08	1.30
	[1.03 – 1.54]	[0.44 – 1.51]	[0.74 – 1.56]	[0.81 – 2.08]
Yes				

+ $p < 0.1$, * $p < 0.05$, ** $p < 0.01$, *** $p < 0.001$

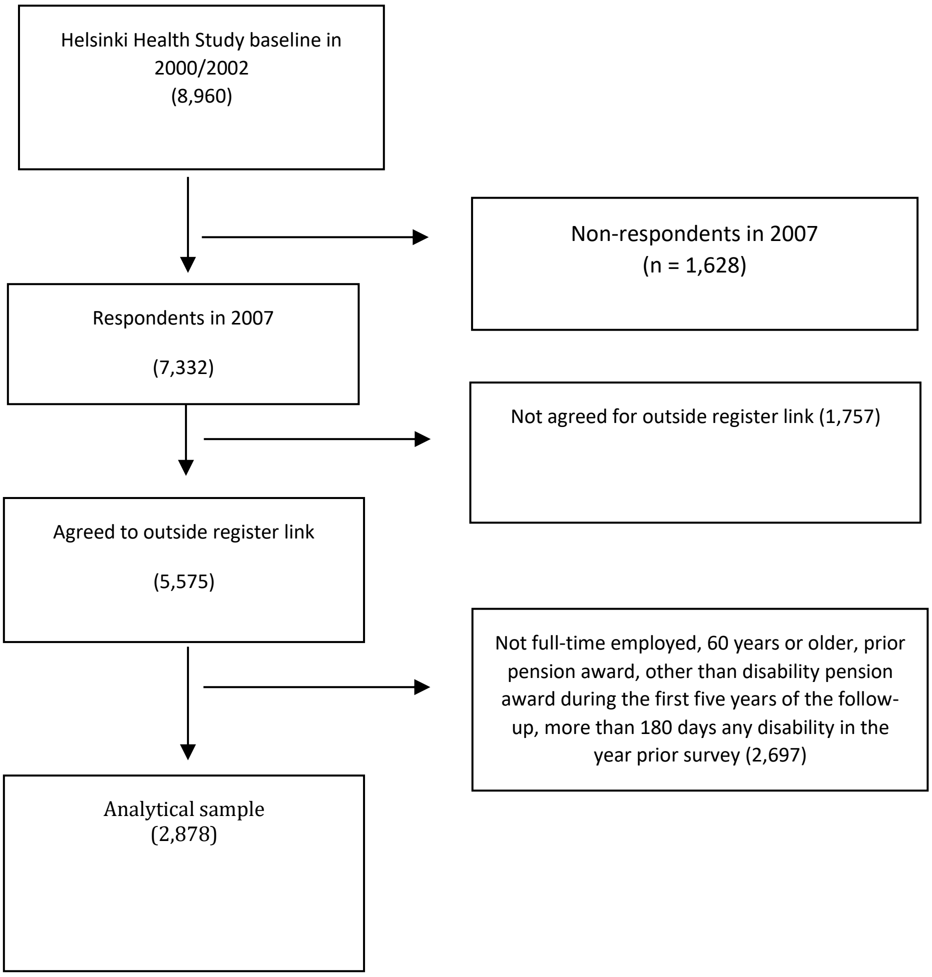

Supplementary Figure S1. Flowchart of the study population

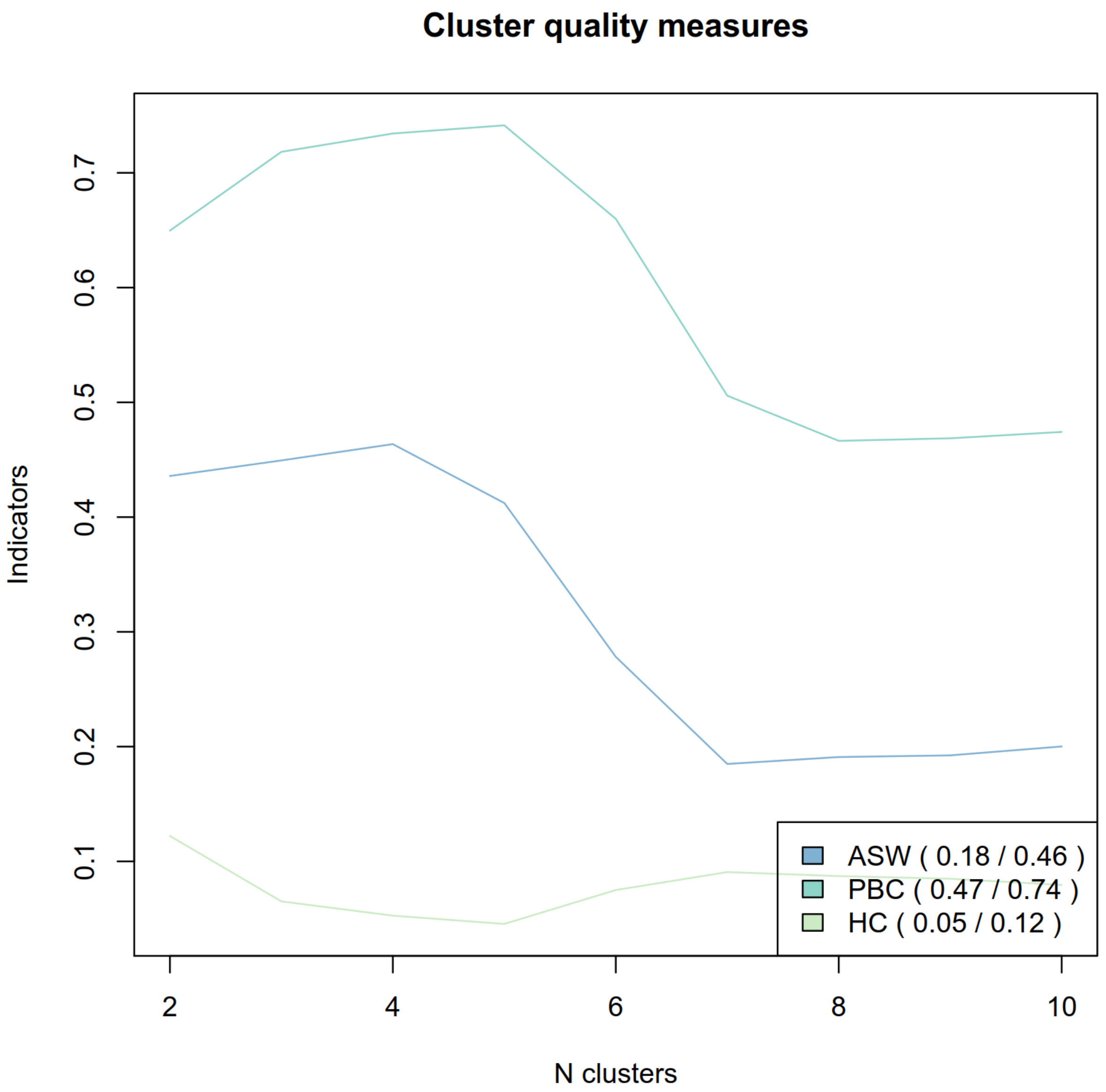

Supplementary figure S2. Cluster quality measures by number of clusters on the horizontal axis. The selected number of clusters N=4. The work disability development was clustered using longest common subsequence similarity measure and Ward's clustering algorithm. Optimal number of clusters was selected based on the cluster quality measures, reasonable group sizes and substantially meaningful interpretation. Note that the fifth cluster was manually formed from those employees without any work disability during the follow-up period and therefore not in this chart. ASW= Average Silhouette Width [max], PBC = Point Biserial Correlation [max], HC = Hubert's C [min].

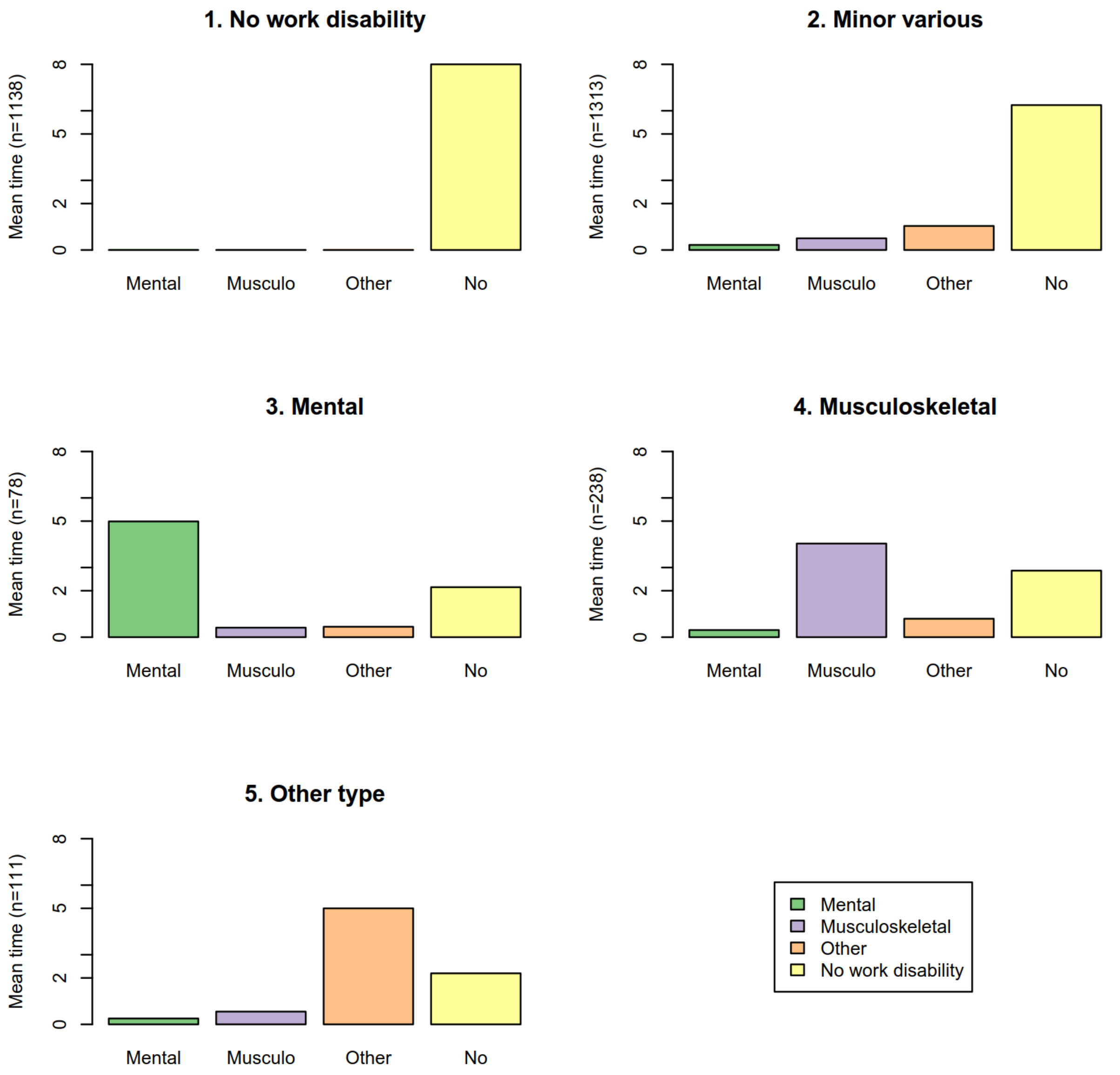

Supplementary Figure S3. Mean time spent on each of the four work disability state (vertical axis, ranging from 0-8) by the identified five work disability clusters. Time unit is years (after submitting the survey). The work disability development was clustered using longest common subsequence similarity measure and Ward's clustering algorithm. Optimal number of clusters was selected based on the cluster quality measures, reasonable group sizes and substantially meaningful interpretation. Note that the 1. no work disability cluster was manually formed from those employees without any work disability during the follow-up period. Yellow = no work disability, purple = Musculoskeletal work disability (M00-M99), green = mental work disability (F00-F99), sand=other work disability.

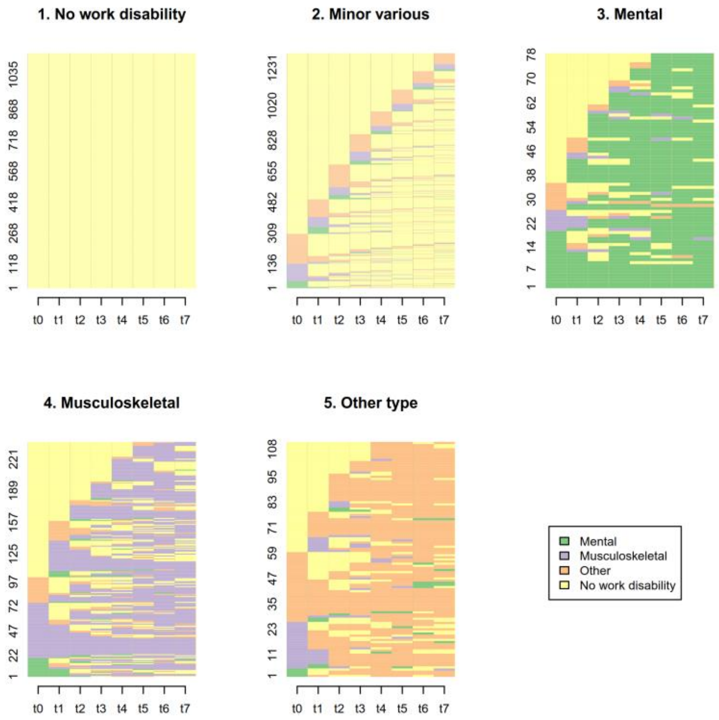

Supplementary Figure S4. All individual sequences in the data shown by the five work disability clusters. The follow-up period is on the horizontal axis and individual sequences on the vertical axis. Individual sequences are ordered within clusters based on the "no work disability state". The work disability development was clustered using longest common subsequence similarity measure and Ward's clustering algorithm. Optimal number of clusters was selected based on the cluster quality measures, reasonable group sizes and substantially meaningful interpretation. Note that the 1. no work disability cluster was manually formed from those employees without any work disability during the follow-up period. Yellow = no work disability, purple = Musculoskeletal work disability (M00-M99), green = mental work disability (F00-F99), sand=other work disability.

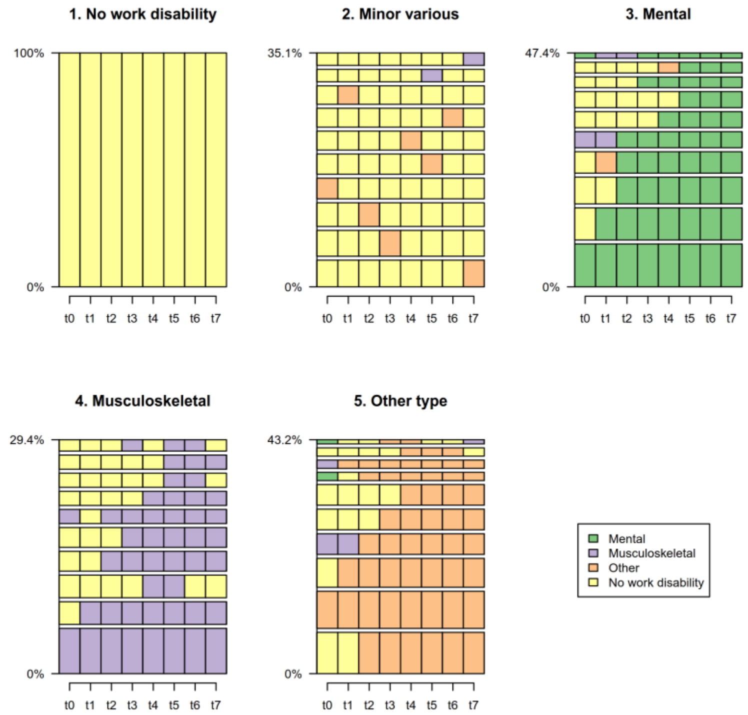

Supplementary Figure S5. The ten most frequent sequences in the data shown by the five work disability clusters. The widths of the bars are proportional to the occurrence of the corresponding sequence in the cluster. The percentages in the top of the vertical axis presents the proportions of the ten most frequent sequences of all sequences in the given cluster. Horizontal axis presents the follow-up period. The work disability development was clustered using longest common subsequence similarity measure and Ward's clustering algorithm. Optimal number of clusters was selected based on the cluster quality measures, reasonable group sizes and substantially meaningful interpretation. Note that the 1. no work disability cluster was manually formed from those employees without any work disability during the follow-up period. Yellow = no work disability, purple = Musculoskeletal work disability (M00-M99), green = mental work disability (F00-F99), sand=other work disability.

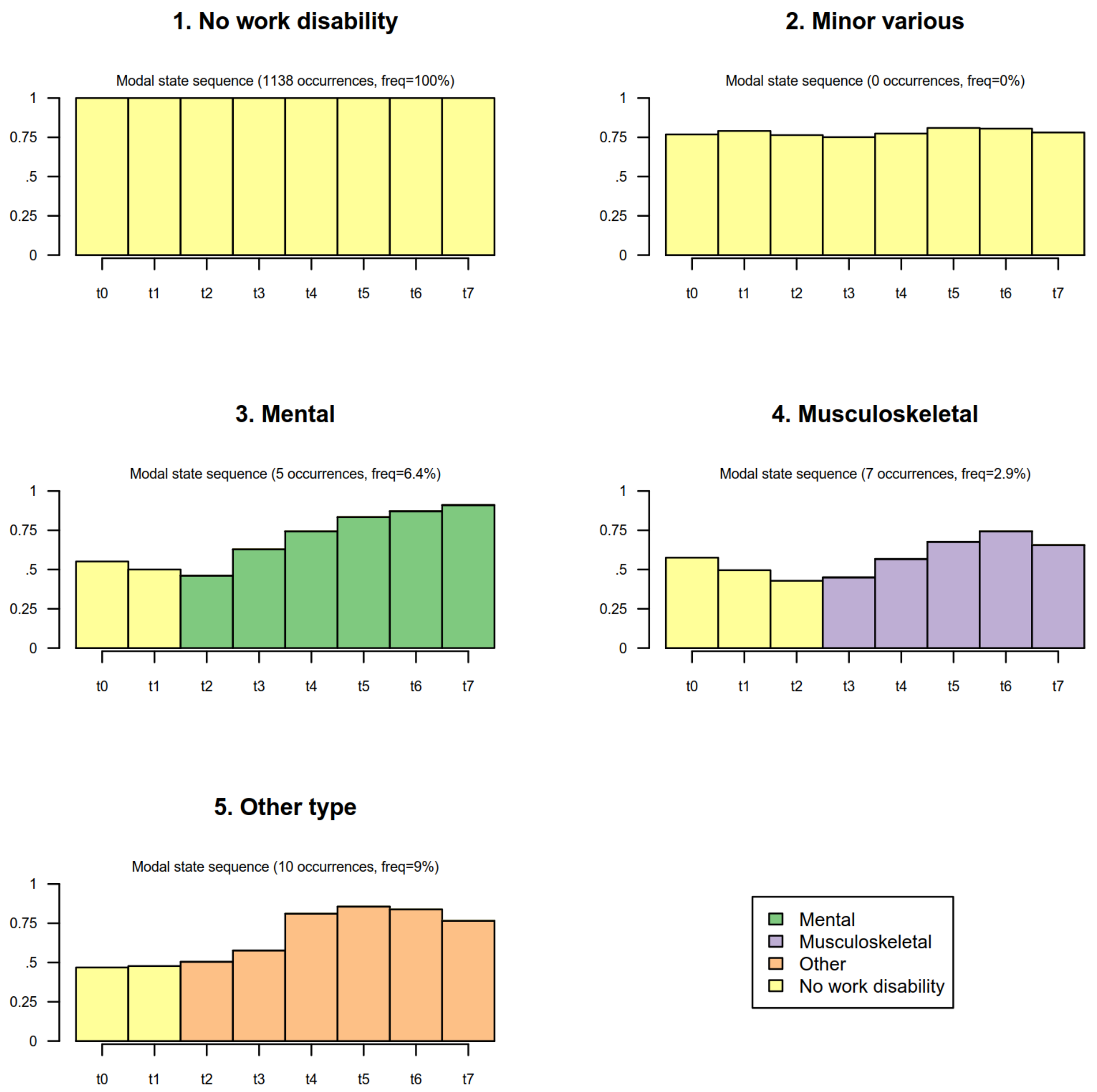

Supplementary Figure S6. The most common (modal) state in each time-point by the identified five work disability clusters. The follow-up period is on the horizontal axis and the vertical axis presents the proportion of this state in the given time unit. The work disability development was clustered using longest common subsequence similarity measure and Ward's clustering algorithm. Optimal number of clusters was selected based on the cluster quality measures, reasonable group sizes and substantially meaningful interpretation. Note that the 1. no work disability cluster was manually formed from those employees without any work disability during the follow-up period. Yellow = no work disability, purple = Musculoskeletal work disability (M00-M99), green = mental work disability (F00-F99),
